# RSM approach for process optimization of the photodegradation of congo red by a novel NiCo_2_S_4_/chitosan photocatalyst

**DOI:** 10.1038/s41598-024-51618-2

**Published:** 2024-01-11

**Authors:** Vishal Gadore, Ashish Kumar Singh, Soumya Ranjan Mishra, Md. Ahmaruzzaman

**Affiliations:** Department of Chemistry, National Institute of Technology Silchar, Silchar, Assam 788010 India

**Keywords:** Pollution remediation, Nanoscale materials

## Abstract

The current study reported a facile co-precipitation technique for synthesizing novel NiCo_2_S_4_/chitosan nanocomposite. The photocatalytic activity of the prepared nanocomposite was evaluated using congo red (CR) dye as a target pollutant. The central composite design was employed to examine the impact of different reaction conditions on CR dye degradation. This study selected the pH, photocatalyst loading, initial CR concentration and reaction time as reaction parameters, while the degradation efficiency (%) was selected as the response. A desirability factor of 1 suggested the adequacy of the model. Maximum degradation of 93.46% of 35 ppm dye solution was observed after 60 min of visible light irradiation. The response to surface methodology (RSM) is a helpful technique to predict the optimum reaction conditions of the photodegradation of CR dye. Moreover, NiCo_2_S_4_/Ch displayed high recyclability and reusability up to four consecutive cycles. The present study suggests that the prepared NiCo_2_S_4_/chitosan nanocomposite could prove to be a viable photocatalyst for the treatment of dye-contaminated wastewater.

## Introduction

Secure and pure water availability has become a serious concern because of the persistent and unacceptable discharge of organic contaminants into waterways from various sources^1,2^. These contaminants are regarded as highly toxic and persistent in the ecology, having an impact on both human health and the environment^3,4^. Numerous sectors, including textile, pharmaceutical, nourishment, beauty aids, plastic, rubber, and paper, use significant quantities of organic dyes to colour their goods^5,6^. When released into the ecosystem, these coloured compounds can harm aquatic life and human health and can be observed even at minimal concentrations^7,8^. Organic dyes such as Congo red (CR) have been recognized as potent human carcinogens by the IARC "International Agency for Research on Cancer" due to their ability to induce tumours in animals^9^. It contaminates water and soil, leading to harmful effects on aquatic organisms and other wildlife.

Many traditional techniques for cleaning wastewater, including biodegradation, electrochemical combustion, and chemical oxidation, are ineffective in removing organic pollutants^9,10^. A heterogeneous photocatalysis method using semiconductor heterojunctions as photocatalysts has been designed to efficiently utilize solar power as a visible source to degrade organic pollutants from water^11^. Recent research has demonstrated that photocatalysis methods are thought to be the most effective and practical methods for eliminating organic matter from wastewater due to their high rate of degradation, reduced generation of secondary pollutants, efficacy, and cost-effectiveness^12,13^. Photocatalysis is the term for the light-induced chemical reaction that generates electron and hole pairs when subjected to sufficiently strong light. Reactive oxygen species (ROS), such as superoxide (^•^O_2_) and hydroxyl (^•^OH) radicals, are generated when these photogenerated electron/hole pairs interact with oxygen and water. These ROS then attack the target pollutants, degrading them into less harmful intermediates and ultimately converting them into carbon dioxide and water^14,15^.

Metal sulfides have recently attracted much interest as possible heterogeneous catalysts for the photodegradation organic molecules. They have become an excellent replacement for conventional catalysts due to their increased surface area, porous structure, better charge transportation property, non-toxicity, and appropriate bandgap energy suitable for visible regions^16^. However, research into their extensive uses for removing contaminants from industrial effluent is still lacking. In addition, many currently available sulfide-based photocatalysts such as ZnS/ZnO^17^, CuS/CdS^18^, MoS_2_^19^, etc., have poor effectiveness restrictions and are only helpful in situations where the amount of pollutants is very low. Due to their superior electrical and redox responses, mixed metal sulphides (MMSs) significantly outperform mono-metal sulphides in photocatalytic performance^20^. Nickel–cobalt sulfide (NiCo_2_S_4_), a significant ternary transition metal sulfide, has recently received great attention as a potential photocatalyst owing to its optimum bandgap, chemical and thermal resistivity, and quantum size impact^21^. NiCo_2_S_4_ has emerged as an effective photocatalyst for water treatment due to its narrow bandgap energy of 1.2–2.4 eV and excellent light harvesting properties^22,23^.

Furthermore, recent research using its high carrier density as a basis demonstrated that NiCo_2_S_4_ is undoubtedly a metal and not a semiconductor substance^24^. However, NiCo_2_S_4_ suffers from a limitation of firstly, faster charge recombination rate and, secondly, agglomeration of nanoparticles during synthesis^25^. Therefore, NiCo_2_S_4_ utilization as a useful catalyst material for visible-light-induced photocatalytic degradation is still challenging. Thus, the effectiveness of NiCo_2_S_4_ for the photodegradation of organic pollutants could be enhanced by forming a nanocomposite, which will prevent its agglomeration and slow down the charge recombination rate^26^.

Biopolymer-doped metal hydroxides, oxides, and sulfides proved to be potential photocatalysts for degrading dyes from water samples. The efficacy of photodegradation is significantly influenced by biopolymers^27,28^. One of the most prevalent polysaccharides, chitosan (CS) [poly-(1 4) 2-amino-2-deoxy-d-glucose], is a cationic biopolymer that is an unprocessed carbohydrate derived from plants and animals. Strong intra- and intermolecular hydrogen interactions are present in this semi-crystalline polymer. In addition to being steady and secure, it is also reactive, biocompatible, and compostable. It is possible to modify chitosan because it contains volatile amino- and hydroxyl groups^29^. Chitosan is widely used in numerous biological and chemical applications because of its biological traits, non-toxicity, ability to fight cancer, antioxidant properties, ability to heal wounds^30^, and its use in water purification^31^.

The present study illustrates the synthesis of novel NiCo_2_S_4_/chitosan (NiCo_2_S_4_/Ch) via a simple co-precipitation technique and its applicability as a photocatalyst for degrading CR from an aqueous stream. To our understanding, it's the first research on the degradation of CR using a NiCo_2_S_4_/chitosan nanocomposite. The prepared photocatalyst showed enhanced photodegradation of CR under visible light. The enhanced degradation efficiency was attributed to incorporating chitosan, as it helps delocalize photoinduced charges. The presence of reactive groups (NH_2_ and OH) on the chitosan surface could be effective in dye molecule adsorption, which enhances dye degradation by NiCo_2_S_4_ nanoparticles^32^. The effect of reaction parameters on the degradation of CR dye was accessed using response to surface methodology (RSM) with a central composite design (CCD).

## Experimentation

### Reagents

Chitosan [C_18_H_35_N_3_O_13_], Nickel (II) nitrate hexahydrate [Ni(NO_3_)_2_**.**6H_2_O], Cobalt (II) nitrate hexahydrate [Co(NO_3_)_2_**.**6H_2_O], Sodium sulfide hydrate [Na_2_S**.**9H_2_O], Acetic acid [CH_3_COOH], Congo red (CR), and distilled water.

The reagents were acquired from Sigma Aldrich and were of excellent analytical quality and used without extra purification. Distilled water was utilized during the experiment.

### Synthesis of NiCo_2_S_4_/Ch nanocomposite

The NiCo_2_S_4_/chitosan was synthesized via a facile precipitation technique. Initially, 100 mg of chitosan and 5 mL acetic acid were mixed in a 200 mL beaker under constant stirring until a clear solution was obtained. Then, 30 mL of distilled water was added to the above solution and stirred for 15 min at 50 °C. Meanwhile, 2 mmol of [Ni(NO_3_)_2_**.**6H_2_O] and 4 mmol of [Co(NO_3_)_2_**.**6H_2_O] were dissolved in 50 mL of distilled water under constant stirring for 15 min. The salt solution was then added dropwise to the beaker containing chitosan, and the resultant mixture was stirred for 30 min at 50 °C. In another beaker, a solution of 8 mmol of [Na_2_S**.**9H_2_O] in 20 mL distilled water was made and added dropwise to the salt solution. A black-coloured precipitate was observed. The resultant mixture was stirred for another 2 h at the same temperature, and the solution was kept for ageing for 24 h. The final black precipitate was centrifuged, washed and kept for drying in an oven at 80 °C for 7 h. The exact process was carried out without chitosan to synthesize pure NiCo_2_S_4_ nanoparticles.

### Characterization

The X-ray diffraction (XRD) spectrum of the samples was taken from an X'PERT powder X-ray diffractometer with Cu k_α_ radiation. The X-ray photoelectron spectrum of NiCo_2_S_4_/chitosan was recorded using a Thermo Fisher Scientific Pvt. Ltd. ESCALAB Xi + X-ray photoelectron spectrometer (XPS). The shape and particle size of the sample were analyzed through a JEOL JEM-2100 PlusElectron high-resolution transmission electron microscope (HRTEM). The morphology and the energy dispersive X-ray analysis (EDAX) of NiCo_2_S_4_/Ch were accessed using a Carl ZEISS SIGMA field emission scanning electron microscope (FESEM). The liquid chromatography-mass spectroscopy (LCMS) spectrum was recorded using a Xevo XS QTof mass spectrometer. Cary 5000 UV–Vis-NIR spectrophotometer was used to record the UV-DRS spectra. The Fourier Transform Infrared (FTIR) spectrum of NiCo_2_S_4_/Ch was recorded using the Perkin Elmer spectrum 100 instrument.

### Photodegradation tests

All the photodegradation tests were carried out at room temperature (33 ± 2 °C) in a wooden cabinet equipped with a Philips 23 W white LED bulb as the source of visible light with a light intensity of 52.13 W/m^2^. The photodegradation reactions were conducted in a 100 mL beaker with 50 mL dye solution placed 10 cm from the LED light. The light intensity was measured using a Lux meter placed at the base of the beaker.

Typically, in an experiment, the beaker containing 50 mL 35 ppm of dye solution and 22 mg of NiCo_2_S_4_/Ch was irradiated under LED light by placing it in a wooden chamber. The reaction cell was kept in the dark at a constant stirring speed for 30 min to attain adsorption–desorption equilibrium before irradiating it for a prescribed time duration. The dye degradation was examined by tracking the decrease in the absorbance of CR dye at λ_max_ = 497 nm using a UV–visible spectrophotometer (GENESYS 10 S UV–Visible spectrophotometer).

The photodegradation efficiency was calculated from the following equation^33^:

1 $$Degradation \, efficiency \left(\%\right)=\left(\frac{{C}_{0}-C}{{C}_{0}}\right) \times 100$$

The kinetics of the photodegradation of CR under optimum conditions was evaluated according to the following equation:

2 $$\mathit{ln}\frac{{C}_{0}}{C}=kt$$

C_0_ and C are the initial and final concentrations at time t of CR dye, respectively, and k is the pseudo-first-order rate constant.

### Experimental design

The effect of the reaction factors such as pH (A), photocatalyst dosage (B), initial dye concentration (C) and time (D) on the photocatalytic activity of NiCo_2_S_4_/Ch catalyst was examined using response to surface methodology (RSM) employing central composite design (CCD). As CR dye is an indicator and is blue in an acidic medium, the study of pH is limited to alkaline conditions. Design Expert Software (DOE) version 13 was used to create the design of the experimental analysis. The RSM is a collection of mathematical and statistical methods that help investigate the effect of individual parameters on the response^34^. These experimental data were fitted to achieve the best degradation efficiency. RSM gives a set of reaction conditions which help examine the effect of single-parameter and multi-parameter interactions and the relationship between operating parameters on the photodegradation rate of CR dye to achieve the optimum conditions^35^. According to the RSM analysis, an experimental design of 30 runs (16-factor points + 8 axial points + 6 replicates) was considered as the empirical model according to the following equation:

3 $$N= {2}^{k}+2k+6$$

N, Number of experimental runs; and k, Number of input variables (parameters).

A quadratic equation predicted the relation between CR photodegradation and the individual factors. The response was predicted using the following equation^36^:

4 $$Y= {\beta }_{0}+ \sum\limits_{i=1}^{k}{\beta }_{i}{X}_{i}+ \sum\limits_{j=1}^{k}{\beta }_{jj}{X}_{j}^{2}+ \sum\limits_{i=1}^{k}\sum\limits_{j=1}^{k}{\beta }_{ij}{X}_{i}{X}_{j}$$

where X_i_ is the coded value of the ith individual parameter, β_0_, β_i,_ and β_ij_ are the equation’s zero, first and second-order coefficients, respectively.

The analysis of variance (ANOVA) and F-test were used to appraise the results, and the coefficients of R^2^ and R^2^_adj_ were used to assess the polynomial model's fitness. A careful comparison between the experimental and predicted outcomes was examined at the conclusion to demonstrate the predicted model's statistical significance.

## Results and discussion

### XRD analysis

**Figure 1:**
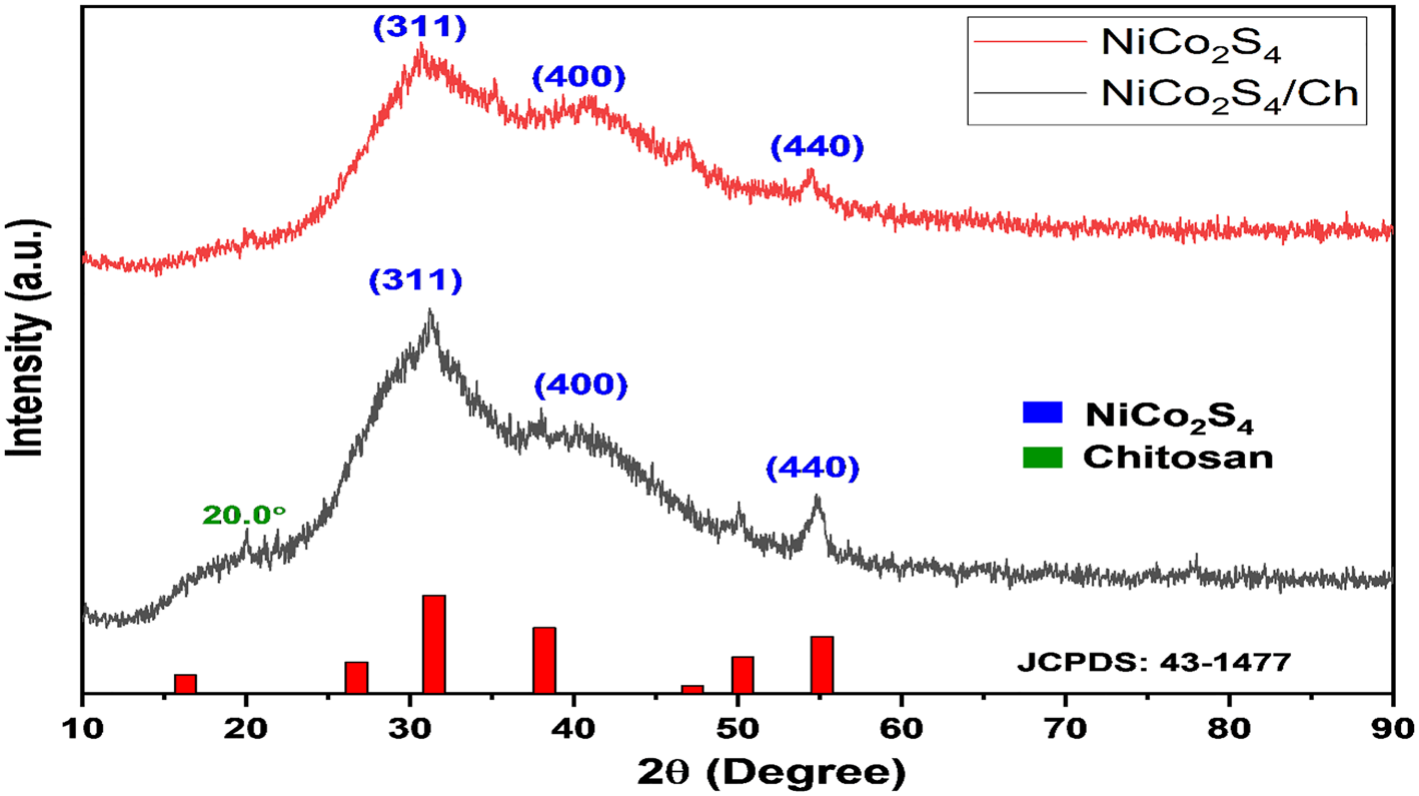
XRD spectrum of NiCo_2_S_4_ and NiCo_2_S_4_/Ch.

The XRD is a useful technique to access the crystal structure of the nanocomposites. The XRD spectra of NiCo_2_S_4_ and NiCo_2_S_4_/Ch are shown in Fig. 1. The XRD spectrum of NiCo_2_S_4_ showed the broad and major diffraction peaks at 2θ = 31.47°, 38.19° and 55.10° could be assigned to (311), (500), and (440) planes of cubic NiCo_2_S_4_, respectively, matching with the JCPDS card No. 43-1477, having lattice parameter a = b = c = 9.417 Å^37^. The broad peaks in the XRD spectrum indicate the amorphous nature of the sample^38^. The XRD spectrum of NiCo_2_S_4_/Ch showed a shift of the peaks to a higher angle because of the distortion in the growth phase of NiCo_2_S_4_ due to the presence of chitosan^39^, and all the peaks corresponding to (311), (500), and (440) planes of cubic NiCo_2_S_4_ could be identified. The sift of the peaks and the appearance of a new peak at 2θ = 20.0° appeared, indicating the incorporation of chitosan in the nanocomposite^40^. The average crystallite size of the NiCo_2_S_4_/Ch nanocomposite was found to be 8.17 nm using Debye–Scherrer's equation.

### Morphological and elemental analysis

**Figure 2:**
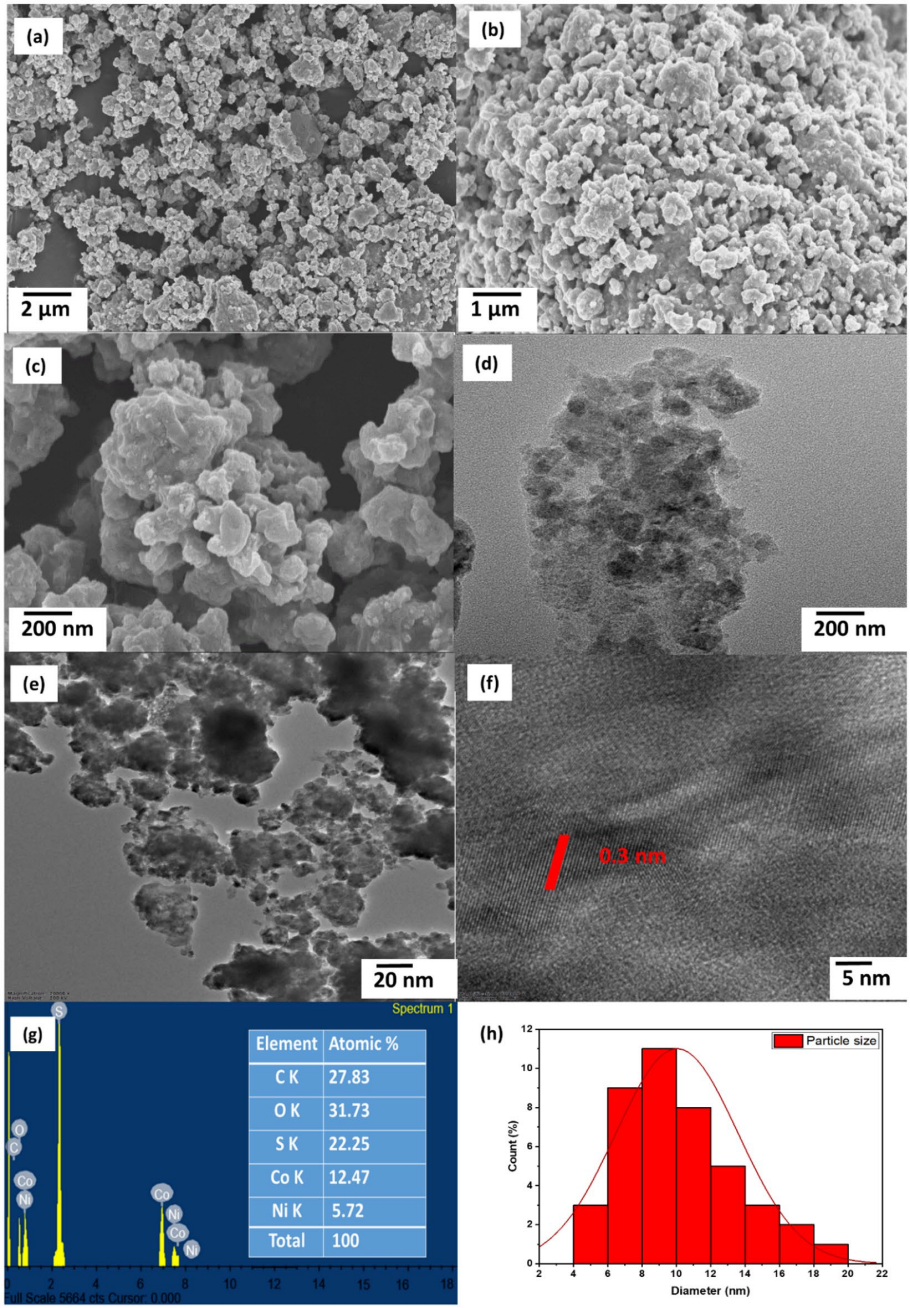
(**a**–**c**) SEM images, (**d**) TEM image, (**e**–**f**) HRTEM images, (**g**) EDAX spectrum and (**h**) size distribution histogram of NiCo_2_S_4_/Ch nanocomposite.

The SEM images of the NiCo_2_S_4_/Ch nanocomposite illustrated in Fig. 2a–c displayed that the surface of the prepared sample was rough due to the dispersion of small spherical NiCo_2_S_4_ nanoparticles over the chitosan surface. The presence of small granular NiCo_2_S_4_ nanoparticles could be seen in the high-resolution SEM images. The TEM images of NiCo_2_S_4_/Ch nanocomposite showed an irregular distribution of NiCo_2_S_4_ nanoparticles over the chitosan (Fig. 2d). Clear lattice planes with lattice fringes of spacing 0.3 nm corresponding to (400) lattice plane of NiCo_2_S_4_ could be identified and marked in the HRTEM images of NiCo_2_S_4_/Ch nanocomposite (Fig. 2e,f). The size distribution histogram was plotted, and the average particle size could be calculated from the histogram seen in Fig. 2h. An average particle of the NiCo_2_S_4_/Ch nanocomposite was observed to be 10.0 nm in accordance with the average crystallite size. The EDAX spectrum (Fig. 2g) revealed the presence of nickel, cobalt, sulfur, oxygen and carbon with relative atomic percentages of 5.72%, 12.47%, 22.25%, 31.73%, and 27.83%. At the same time, a higher amount of carbon and oxygen is due to the chitosan^41^.

### XPS analysis

**Figure 3:**
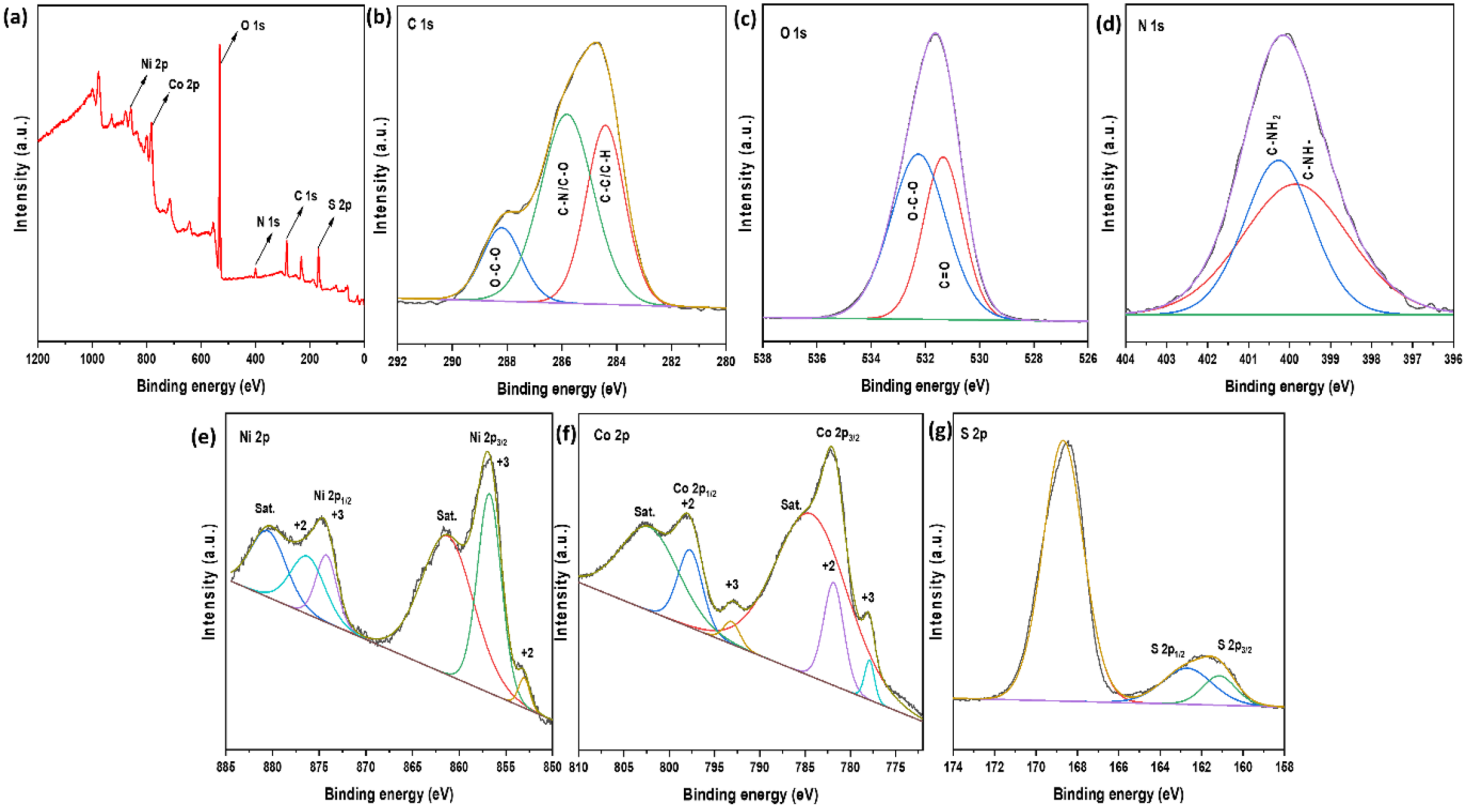
(**a**) XPS survey, short scan of (**b**) C 1s, (**c**) O 1s, (**d**) N 1s, (**e**) Ni 2p, (**f**) Co 2p and (**g**) S 2p.

The oxidation states and elemental composition of the NiCo_2_S_4_/Ch nanocomposite were analyzed through XPS. The survey spectrum of NiCo_2_S_4_/Ch showed the presence of Ni 2p, Co 2p, S 2p, C 1s, O 1s and N 1s as expected (Fig. 3a). As seen in Fig. 3b, the short scan of C 1s showed a triplet of peaks appearing at 284.4 eV, 258.8 eV and 288.2 eV. The peaks at lower binding energies, i.e. 284.4 eV and 258.8 eV, are attributed to the carbon as in C–C/C–H and C–O/C–N bonds, respectively^42^. The higher binding energy peak at 288.2 eV shows the presence of sp^2^ hybridized carbon in C=O or C–O–C bonds^43^. The short scan spectrum of O 1s (Fig. 3c) showed a doublet of peaks appearing at 531.3 eV assigned to C=O and 532.2 eV due to C–O–C bonds^44^. A shift in the binding energies to greater values for C 1s and O 1s in NiCo_2_S_4_/Ch could be attributed to the insertion of NiCo_2_S_4_ nanoparticles, which increased the electron density near C and O, causing a shift in the binding energies to higher values^45^. The core-level spectrum of N 1s is shown in Fig. 3d. Here, a doublet of peaks was also observed, similar to the O 1s spectrum. The peaks appeared at 399.8 eV and 400.2 eV, which could be assigned to C–NH and C–NH_2_ groups, respectively^46^. Figure 3e illustrates the deconvoluted spectrum of Ni 2p, showing the presence of a pair of doublets and satellite (Sat.) peaks. The 856.5 eV and 874.5 eV peaks correspond to Ni 2p_3/2_ and Ni 2p_1/2_, respectively, characteristic of the + 3 oxidation state of Ni atoms, while the peaks at 853.0 eV and 876.2 eV are distinctive of Ni^+2^ atoms, indicting the co-existence of Ni^+2^ and Ni^+3^ ions in NiCo_2_S_4_/Ch composite^47^. Figure 3f shows the deconvoluted short scan of Co 2p, which is quite similar to Ni 2p spectra. The strong peaks at 782.0 eV could be assigned to Co 2p_3/2_, and the peak at 798.1 eV corresponds to Co 2p_1/2_, indicating the presence of Co^+2^ ions, while the peaks at 778.0 eV and 793.1 eV are characteristic of Co^+3^ ions which is also present in small amounts, suggesting the co-existence of Co^+2^ and Co^+3^ ions in the composite^48^. The peaks at 784.5 eV and 802.5 eV are the satellite peaks of Co 2p. The typical short scan spectra of S 2p showed two main peaks and a satellite peak (Fig. 3g). The peaks at 161.2 eV and 162.7 eV are the characteristic peaks of S 2p_3/2_ and S 2p_1/2_, respectively, attributed to the − 2 oxidation state of sulfur^49,50^.

### Functional group analysis

**Figure 4:**
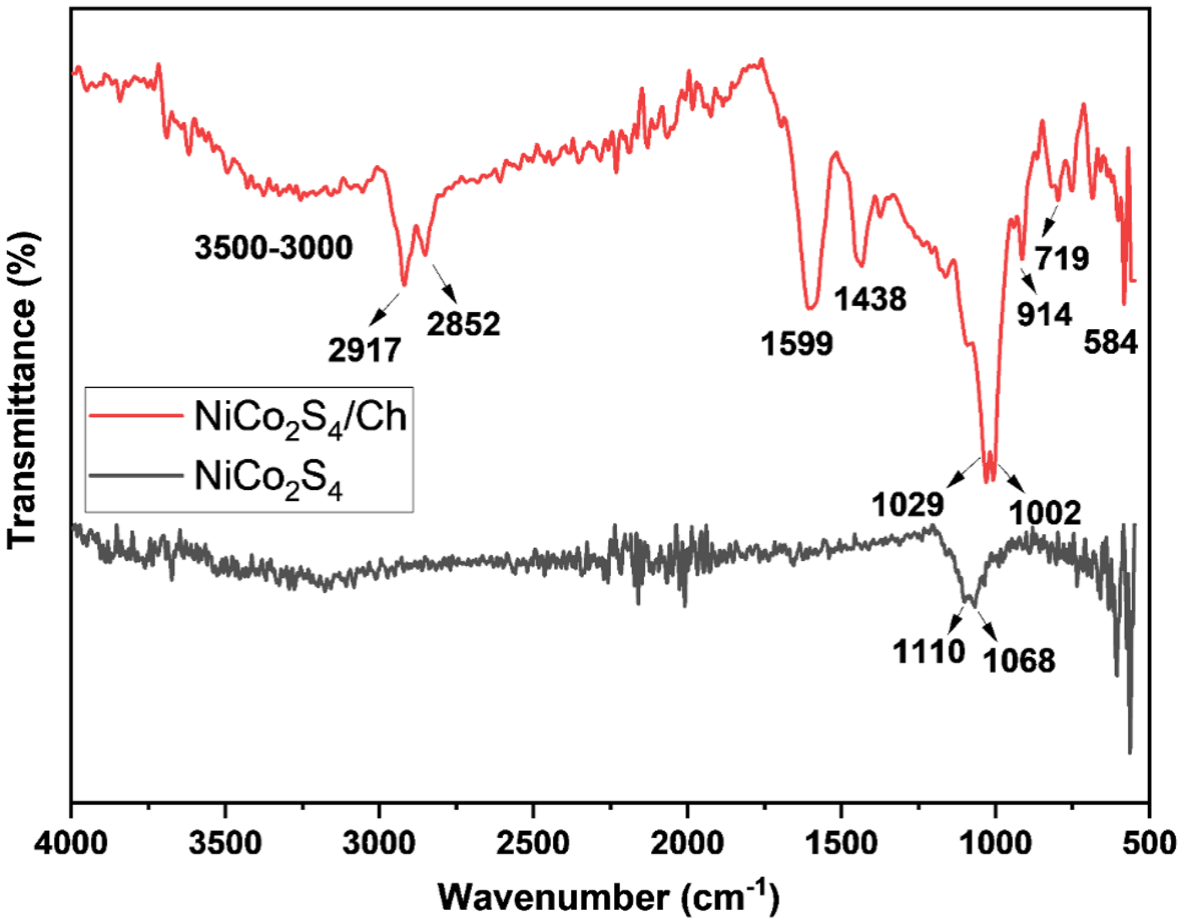
FTIR spectrum of pure NiCo_2_S_4_ and NiCo_2_S_4_/Ch nanocomposite.

The presence of the chitosan and the surface functional group were also analyzed using FTIR spectroscopy. The FTIR spectrum of NiCo_2_S_4_ and NiCo_2_S_4_/Ch nanocomposite is illustrated in Fig. 4. Pure NiCo_2_S_4,_ being an inorganic compound, showed two peaks at 1110 and 1068 cm^−1^ attributed to C–O stretching in adsorbed carbon dioxide. A wide peak in the region 3500–3000 cm^−1^ occurred due to the stretching of O–H and N–H bonds of chitosan overlapping in the same region. Two small peaks at 2917 cm^−1^ and 2852 cm^−1^ are the characteristic stretching bands of C–H groups present in chitosan^51^. The occurrence of C=O bond stretching is indicated by a broad and strong peak at 1599 cm^−1^. The peak at 1438 cm^−1^ was ascribed to the bending vibrations of CH_2_ groups. A strong band between 1029 and 1002 cm^−1^ corresponds to the stretching of C–O bonds. The bands at 914 cm^−1^ and 719 cm^−1^ indicate the out-of-plane bendings of C–H groups. All these bands in the FTIR spectrum of NiCo_2_S_4_/Ch nanocomposite agree with the previous literature and confirm the existence of chitosan in the prepared material^52^. Meanwhile, a small band at 584 cm^−1^ arises due to metal–oxygen stretchings^53^.

### Optical properties

**Figure 5:**
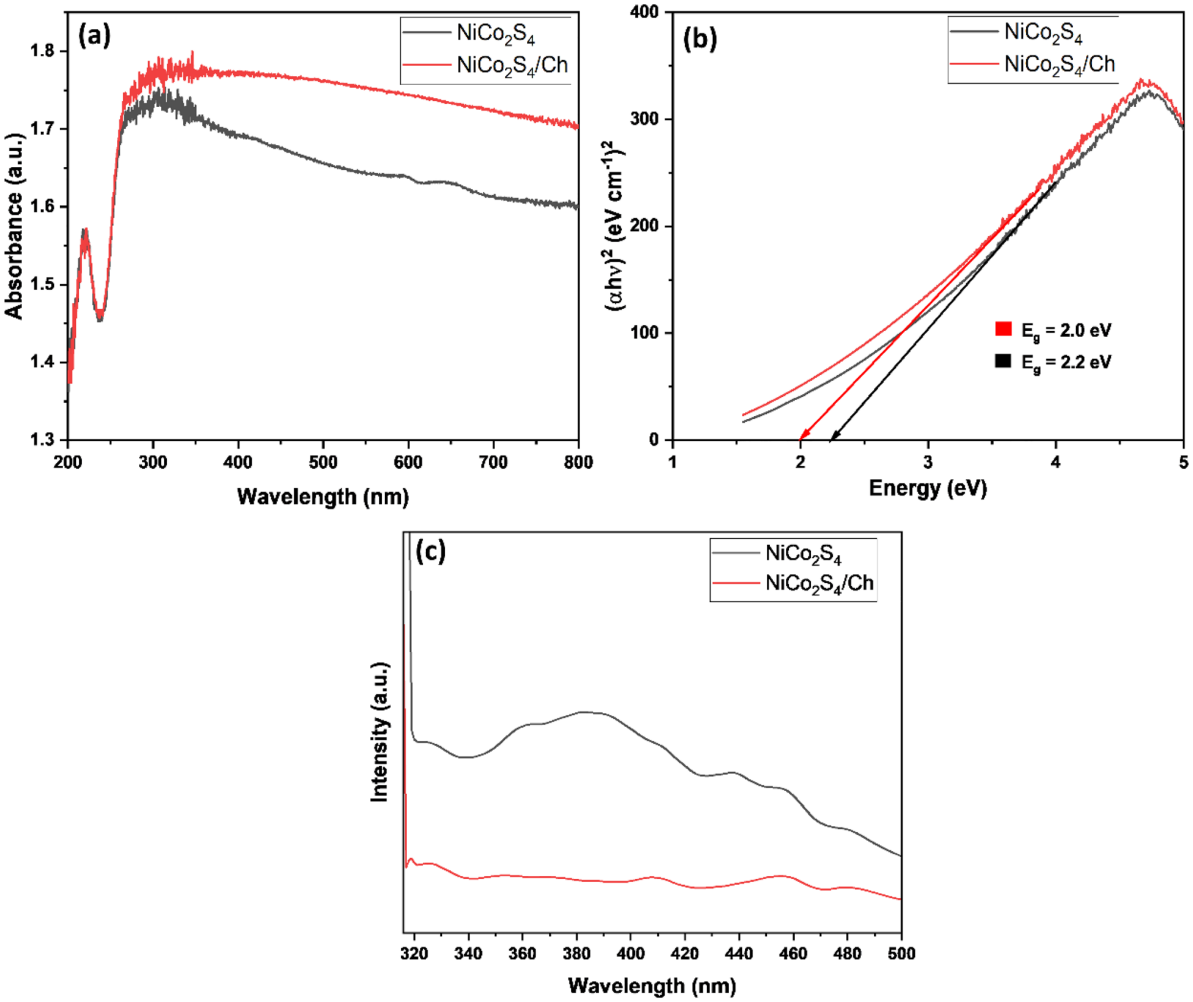
(**a**) The absorption spectrum, (**b**) Tauc's plot, and (**c**) PL spectrum of NiCo_2_S_4_ and NiCo_2_S_4_/Ch.

The optical and light harvesting behaviour of NiCo_2_S_4_ nanoparticles and NiCo_2_S_4_/Ch nanocomposite were investigated using UV-DRS. The absorption spectra of NiCo_2_S_4_ nanoparticles and NiCo_2_S_4_/Ch nanocomposite are shown in Fig. 5. Both the samples showed maximum absorption in the range of 300–310 nm. However, NiCo_2_S_4_/Ch nanocomposite showed higher absorption intensity compared to pure NiCo_2_S_4_ nanoparticles, indicating improved light harvesting properties. Additionally, both the samples showed strong absorption in the whole UV–visible region, suggesting the excellent light absorption tendency of NiCo_2_S_4_. The band gap of the photocatalyst was calculated using Tauc's plot according to the following equation:

5 $${\left(\alpha h\nu \right)}^{n} = A\left(h\nu -{E}_{g}\right)$$

here α, Coefficient of absorption; h, Planck's constant; ν, Frequency of light; A, Constant; E_g_, Optical bandgap of the material, and n is ½ for indirect and 2 for direct allowed transitions, respectively.

NiCo_2_S_4_ nanoparticles displayed a bandgap of 2.2 eV, which is in good agreement with the formerly published studies^54^. The bandgap reduced from 2.2 to 2.0 eV for the NiCo_2_S_4_/Ch nanocomposite, possibly due to increased particle size after forming the nanocomposite^55^. Additionally, a low bandgap of the material indicates high light-harvesting abilities.

One useful method for determining the visible light photocatalytic activity of the produced nanocomposites is photoluminescence (PL) spectroscopy. The higher intensity of the PL peak indicates a faster rate of electron–hole recombination and a lower photocatalytic activity, while a low-intensity PL peak has a slower charge recombination rate and a higher photocatalytic activity^56^. In the PL spectrum, pristine NiCo_2_S_4_ displayed the maximum intensity, indicating the highest charge recombination rate (Fig. 5c). When chitosan and NiCo_2_S_4_ are combined, the PL peak's intensity drops. Higher photocatalytic activity requires a substantially lower charge recombination rate, which is indicated by the drop in PL intensity^57^. As expected, the NiCo_2_S_4_/Ch nanocomposite displayed the highest photocatalytic activity and the lowest intensity in the PL spectrum, indicating the slowest charge recombination rate.

### Optimization of CR degradation using RSM

RSM was used to examine how response factors affected CR's degradation rate. The experimental design was formulated by CCD to attain maximum degradation efficiency of CR in aqueous solutions by adjusting the reaction parameters. CCD formulated an experimental array of 30 runs, and the predicted and actual responses, along with the four reaction parameters, i.e. pH (A), catalyst dosage (B), initial dye concentration (C) and reaction time (D), are shown in Table 1. The predicted equation of CR degradation was as follows:

6 $$ \begin{aligned} {\text{Degradation}}\left( \% \right) & = {93}.{64} + 0.{\text{9175A}} + {1}.{\text{51B}} - {2}.{\text{17C}} + {6}.{\text{69D}} + 0.{\text{3313AB}} - 0.{17}00{\text{AC}} \\ & \quad - 0.0{\text{787AD}} - 0.{\text{2962BC}} - 0.{\text{5225BD}} - 0.{\text{3762CD}} - {1}.{\text{93A}}^{{2}} - {2}.{\text{13B}}^{{2}} - {1}.{\text{67C}}^{{2}} - {11}.{\text{63D}}^{{2}} \\ \end{aligned} $$

**Table 1:** The effect of input parameters used in the modelling with actual and predicted values for CR dye degradation (%).

Runs	pH	Catalyst dosage (mg)	Dye concentration (ppm)	Time (min)	Degradation efficiency (%)		Residual
					Experimental	Predicted	
1	8	24	40	45	66.76	68.19	− 1.43
2	9	22	35	60	93.46	93.46	0
3	8	20	30	75	82.55	83.37	− 0.8183
4	9	18	35	60	82.31	81.92	0.39
5	8	20	40	45	65.17	65.38	− 0.2117
6	10	20	40	45	66.34	66.37	− 0.0317
7	10	20	30	45	69.47	69.7	− 0.2333
8	7	22	35	60	85.13	83.9	1.23
9	9	22	45	60	83.46	82.45	1.01
10	9	22	35	60	93.46	93.46	0
11	10	20	40	75	79.41	79.89	− 0.4767
12	9	26	35	60	87.56	87.95	− 0.39
13	8	24	40	75	80	79.93	0.0733
14	9	22	35	60	93.46	93.46	0
15	10	20	30	75	86.31	84.72	1.59
16	9	22	35	60	93.46	93.46	0
17	10	24	30	75	88	87.95	0.0517
18	10	24	40	75	82	81.93	0.0733
19	9	22	35	60	93.46	93.46	0
20	8	20	30	45	68.12	68.03	0.0867
21	9	22	35	60	93.46	93.46	0
22	10	24	30	45	75.86	75.02	0.8417
23	11	22	35	60	86.33	87.56	− 1.23
24	8	20	40	75	78.53	79.21	− 0.6817
25	10	24	40	45	71.16	70.5	0.6583
26	8	24	30	45	72.34	72.02	0.3167
27	9	22	35	60	93.46	93.46	0
28	8	24	30	75	85.46	85.27	0.1917
29	9	22	25	60	90.11	91.12	− 1.01
30	9	22	35	60	93.46	93.46	0

The equation above represents the combined effect of several factors on the CR degrading efficiency (%). Contrarily, the response is affected favourably and unfavourably by the coefficients with positive and negative values, respectively.

**Figure 6:**
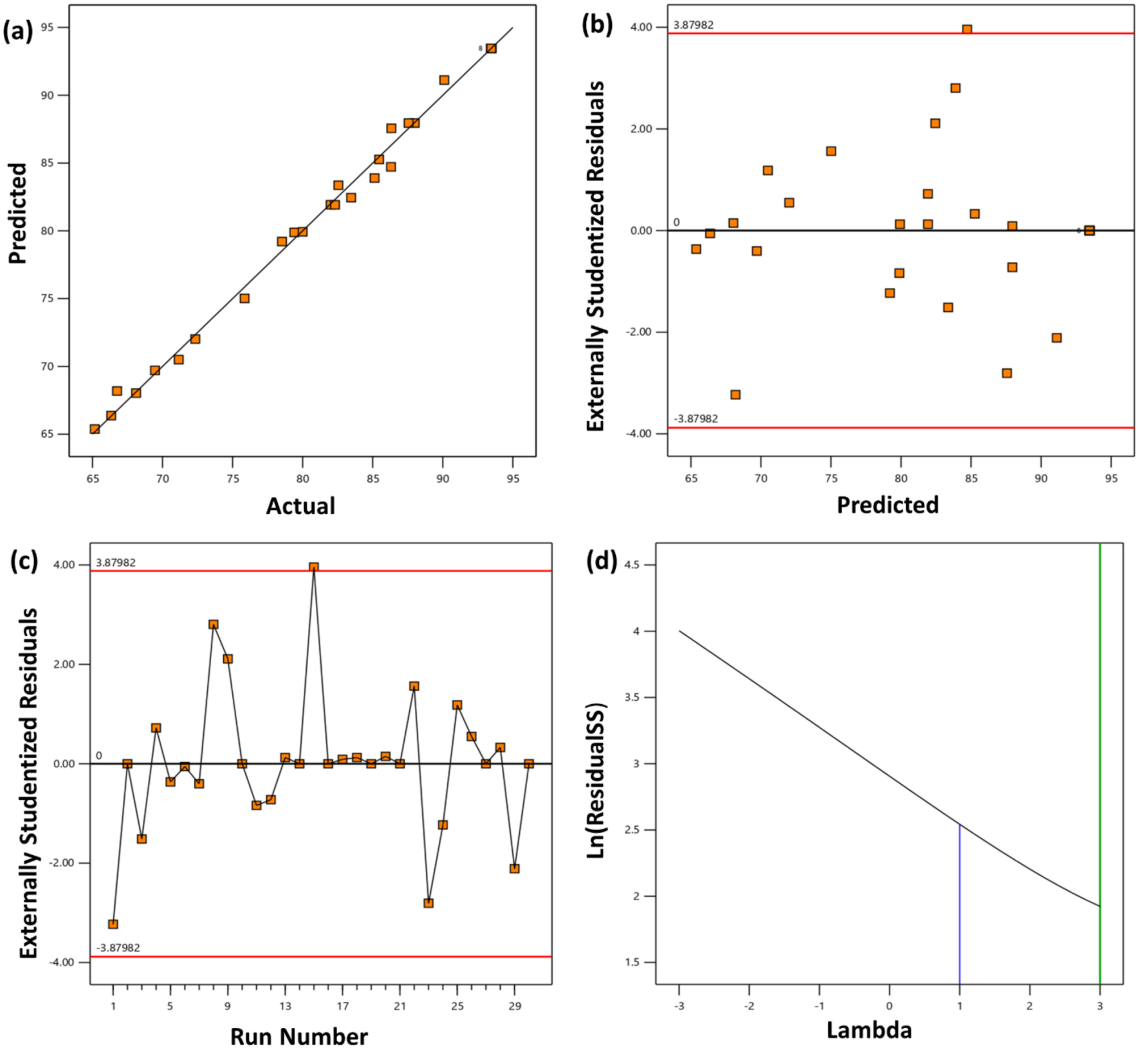
(**a**) Predicted vs actual diagram, (**b**) residuals vs predicted diagram, (**c**) residuals vs run number and (**d**) Box-cox plot for the power transformations for the degradation of CR.

ANOVA was used extensively to examine the impact of the constructed RSM model on the CR degradation efficiency^58^. The ANOVA results and the summary of the quadratic model are given in Table 2. As seen in Table 2, a low *P*-value (< 0.0001), an F-value of 219.11, and a high sum of square values of 2600.50 indicated that the quadratic model is the most suitable. The results reproducibility and the model's implication are generally assessed based on the coefficient of variance (CV) percentage. A CV % ranging between 0.5 and 13.5% is considered an optimum value. Here, a low CV value of just 1.11% indicated a high reproducibility of the proposed quadratic model. Furthermore, a higher value of correlation coefficients (R^2^ = 0.995, Adj R^2^ = 0.990 and Pred R^2^ = 0.961) indicated a well-fitted model^59^. The adequate precision of 43.12 for the degradation of CR shows a good response. A ratio larger than 4 is preferable, showing that the model's mean can deliver the expected performance^60^. The linear fit of actual vs predicted values for CR degradation (R^2^ = 0.995) is shown in Fig. 6a. The plot of residuals vs experimental runs (Fig. 6b,c) depicted all residuals are within ± 4%, indicating the model can predict a good response. The Box-cox plot (Fig. 6d) for power transforms showed a lambda value of 1, demonstrating that the response does not require transformation for the degradation of CR. These results confirm that this model could be used to predict the degradation efficiency of CR dye.

**Table 2:** Results of analysis of variance of the degradation of CR by NiCo_2_S_4_/Ch nanocomposite.

Source	Sum of squares	Degree of freedom (Df)	Mean square	F-value	*P*-value	
Model	2600.5	14	185.75	219.11	< 0.0001	Significant
A-pH	20.2	1	20.2	23.83	0.0002	
B-Catalyst dosage	54.54	1	54.54	64.34	< 0.0001	
C-Dye conc	112.84	1	112.84	133.11	< 0.0001	
D-Time	716.1	1	716.1	844.72	< 0.0001	
AB	1.76	1	1.76	2.07	0.1707	
AC	0.4624	1	0.4624	0.5455	0.4716	
AD	0.0992	1	0.0992	0.117	0.737	
BC	1.4	1	1.4	1.66	0.2176	
BD	4.37	1	4.37	5.15	0.0384	
CD	2.27	1	2.27	2.67	0.1229	
A^2^	95.6	1	95.6	112.78	< 0.0001	
B^2^	116.28	1	116.28	137.17	< 0.0001	
C^2^	71.29	1	71.29	84.09	< 0.0001	
D^2^	825.13	1	825.13	973.34	< 0.0001	
Residual	12.72	15	0.8477			
Lack of Fit	12.72	8	1.59			
Pure error	0	7	0			
Cor total	2613.22	29				

Quadratic summary statics response	R^2^	Adj R^2^	Pred R^2^	Std. Dev	CV %	Adq precision
	0.995	0.990	0.961	0.920	1.11	43.127

**Figure 7:**
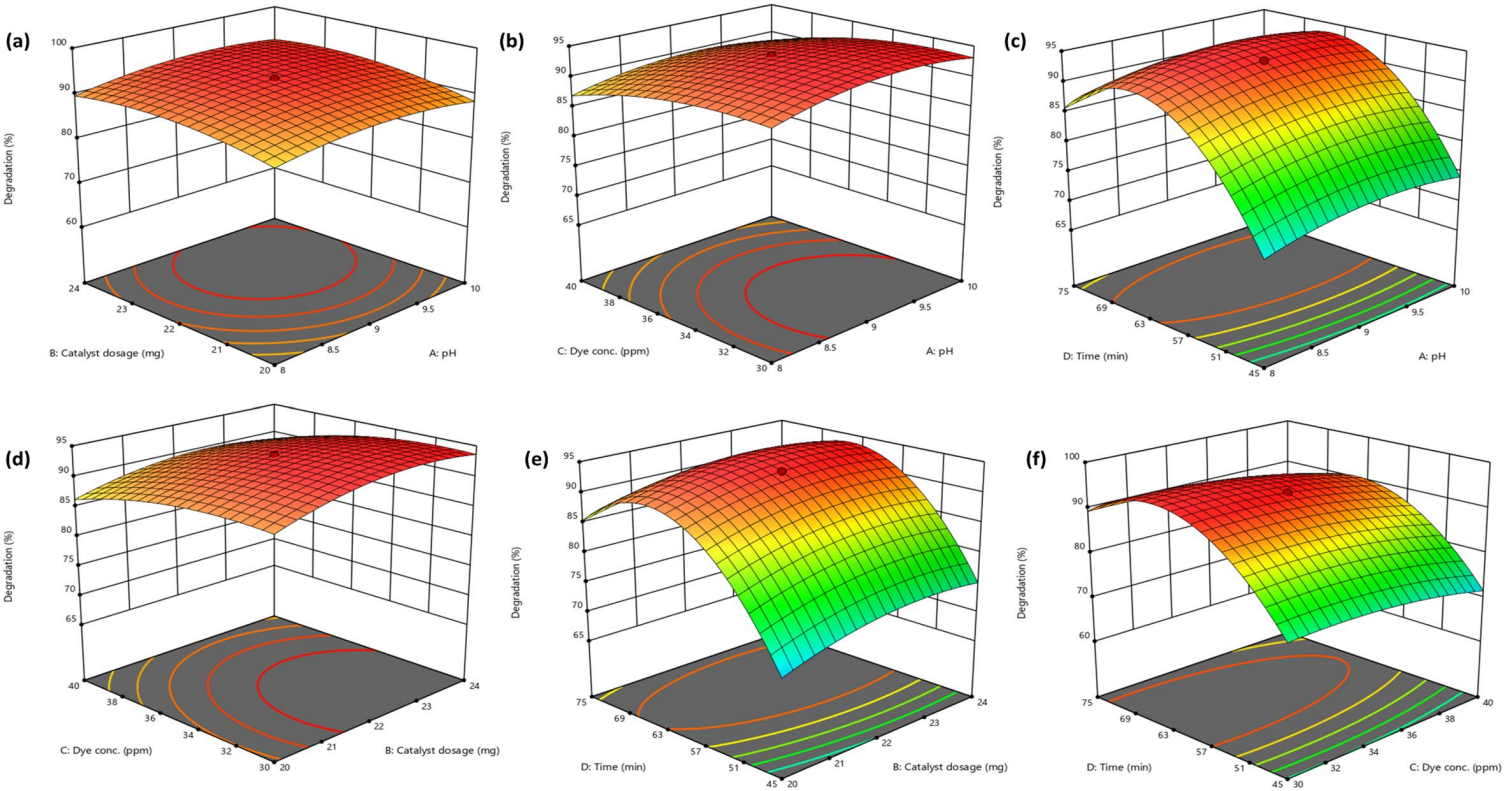
3D surface graphs for the relation between (**a**) pH and catalyst dosage, (**b**) pH and dye concentration, (**c**) pH and time, (**d**) catalyst dosage and dye concentration, (**e**) catalyst dosage and time and (**f**) dye concentration and time.

The relation between various independent factors on the degradation of CR was assessed by developing 3D response surfaces. The results of the interactions among the four parameters are shown in Fig. 7. Figure 7a shows the relation between the pH and catalyst dosage. The degradation process is not considerably impacted by pH. However, the degradation efficiency increases with increasing photocatalyst dosage and starts to decline above the optimum value. The upsurge in the photocatalyst dosage instigated a temporary rise in degradation efficiency brought on by the more active sites. However, the decline in the degradation efficiency of the dye was due to the increased opacity of the solution hindering the photons from reaching the catalyst's surface^61^. Figure 7b illustrates the relationship between pH and dye concentration affecting the rate of CR degradation. A low dye concentration causes a higher degradation rate. The plot of pH and time relating to degradation efficiency shows an initial increase in the degradation, which slumps after the optimum time duration (Fig. 7c). Figure 7d shows the relation between catalyst dosage and dye concentration. The degradation increases at decreasing CR concentration and increasing photocatalyst loading. The relation of catalyst dosage and dye concentration with time is illustrated in Fig. 7e,f. The photodegradation efficiency increases with increasing catalyst dosage and decreasing dye concentration. Similar trends were also observed in various studies investigating the photodegradation of dyes^62,63^. Optimization of these parameters is essential to maximize degradation efficiency and reduce the time and cost of operation. Therefore, in order to get the highest CR dye degradation efficiency, all four parameters were optimized.

**Figure 8:**
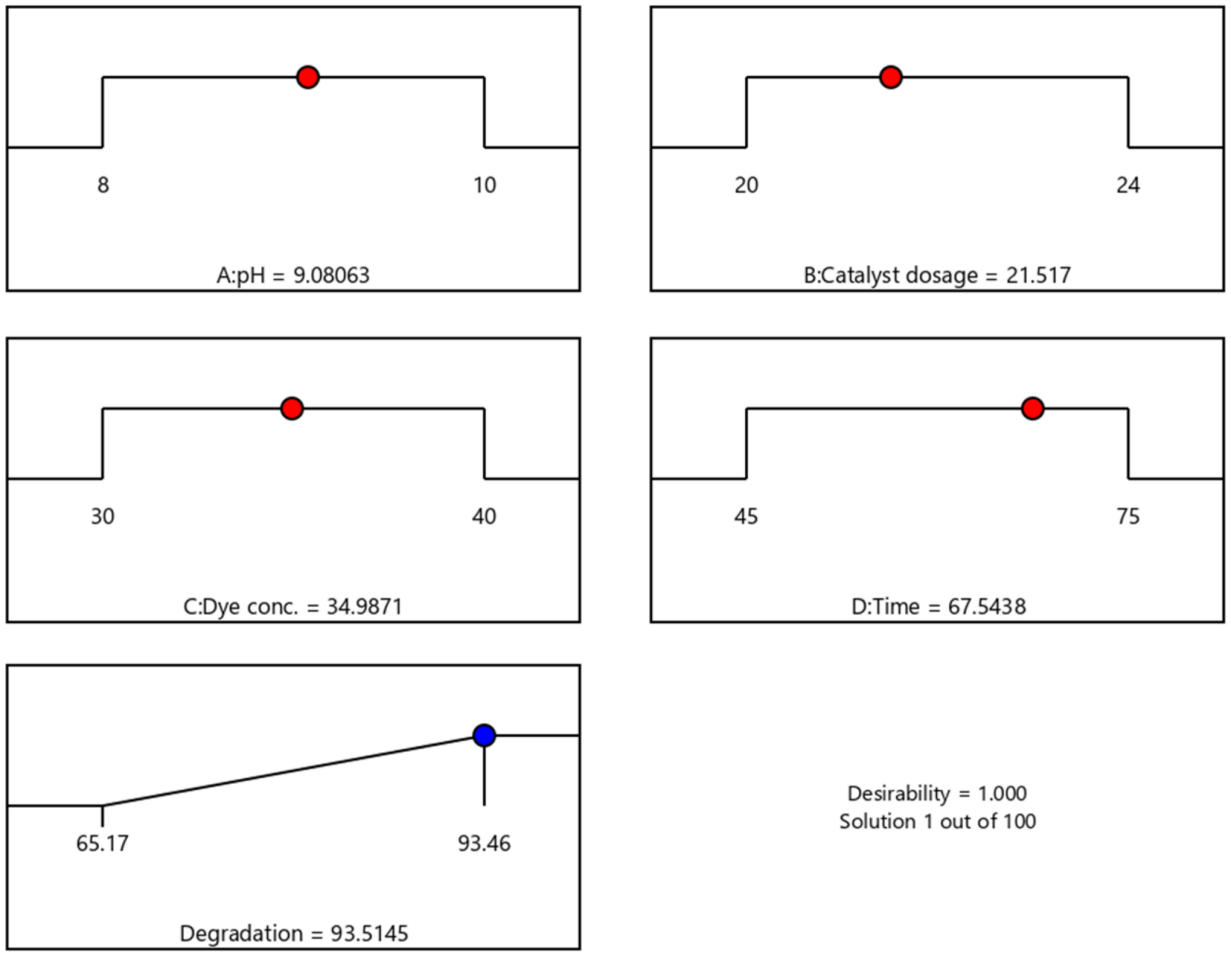
The desirability under optimum conditions.

The RSM has predicted the optimum pH to be 9, catalyst dosage of 21.5 mg, CR concentration of 35 ppm and radiation time of 67 min to attain a maximum degradation efficacy of 93.51% with a desirability value of 1.00. The NiCo_2_S_4_/Ch nanocomposite was shown to be a potential photocatalytic material for the degradation of CR by the desirability factor of 1.00. The optimum value of the independent factor and the desirability value is illustrated in Fig. 8. The actual degradation efficiency of CR was observed to be 93.46% according to run number 16, which is fairly consistent with the value predicted (93.51%). Meanwhile, pure NiCo_2_S_4_ nanoparticles showed 60.23% degradation under similar conditions. The results show the RSM model's significance for optimizing the reaction conditions for CR degradation.

**Figure 9:**
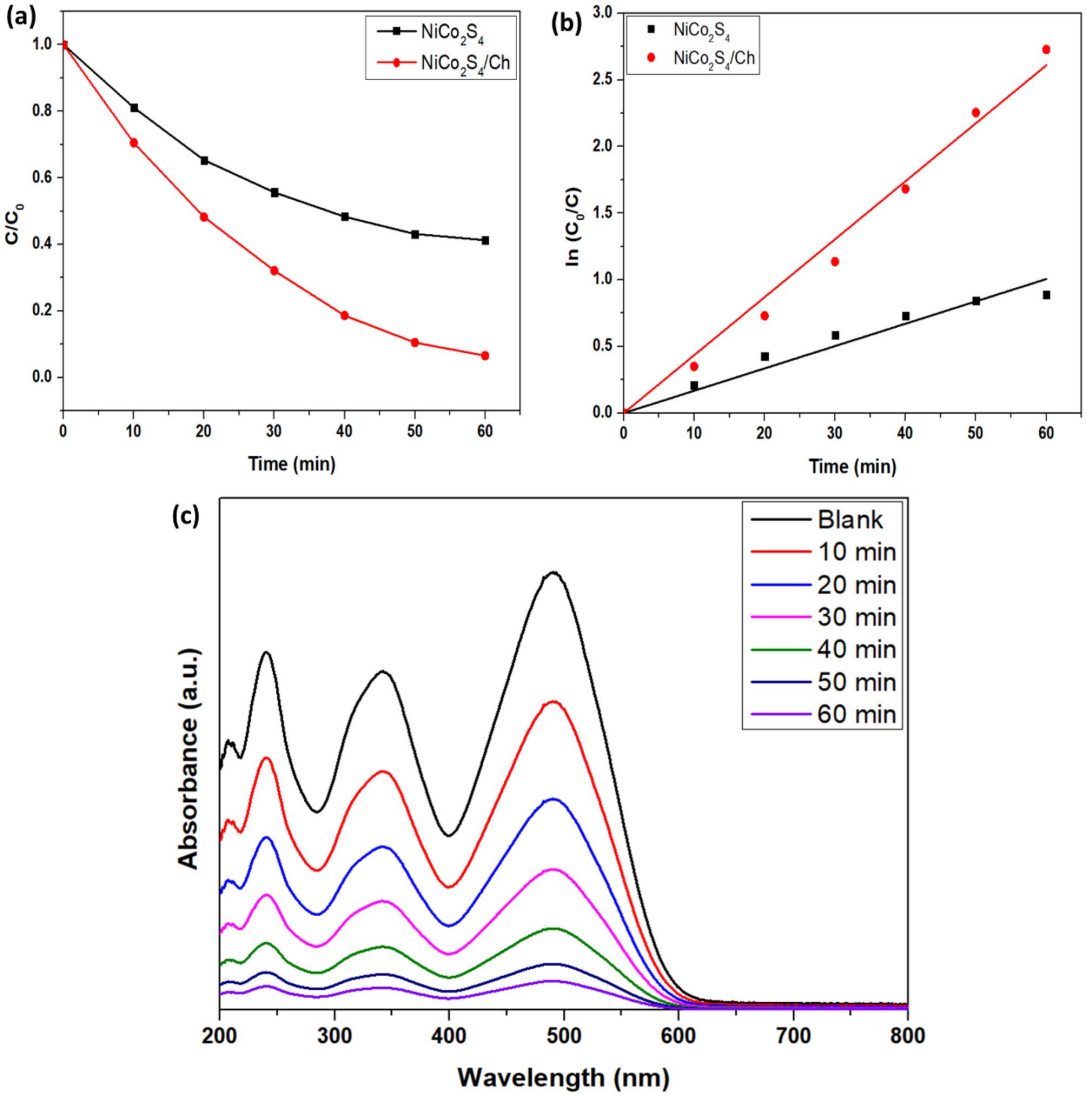
(**a**) Profiles of degradation (**b**) Kinetics and the degradation spectrum of CR dye.

**Figure 10:**
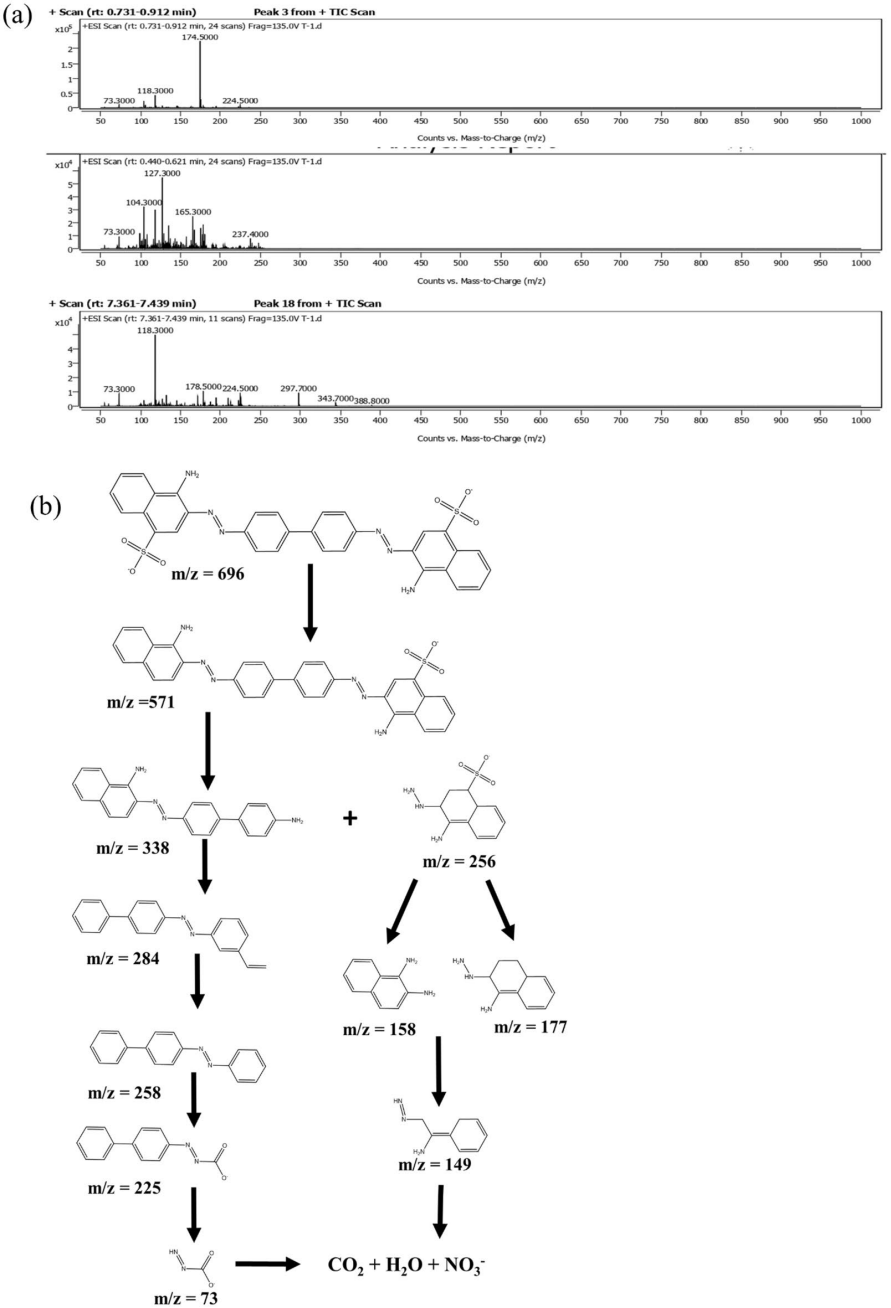
(**a**) Mass spectra of the photodegradation of CR at the intermediate stage and (**b**) plausible mechanism of the degradation of CR dye.

The photodegradation experiment of CR dye was carried out at pH of 9, initial dye concentration of 35 ppm and catalyst dosage of 22 mg, and the degradation kinetics was plotted. Figure 9 shows the profile of the photodegradation of CR and the kinetics. The degradation of CR followed a pseudo-first-order model with a rate constant of 0.043 min^−1^. The synthesized NiCo_2_S_4_/Ch has a higher photodegradation rate than pure NiCo_2_S_4_ nanoparticles. The photocatalytic degradation of CR proceeds via the formation of aromatic intermediate products detected by the HRLCMS, as shown in Fig. 10. The attack of electrons, hydroxyl radicals and the addition of protons formed the intermediates. A plausible mechanism of the photocatalytic degradation pathway of CR dye based on the LCMS analysis is shown in Fig. 10b.

### Scavenging tests

**Figure 11:**
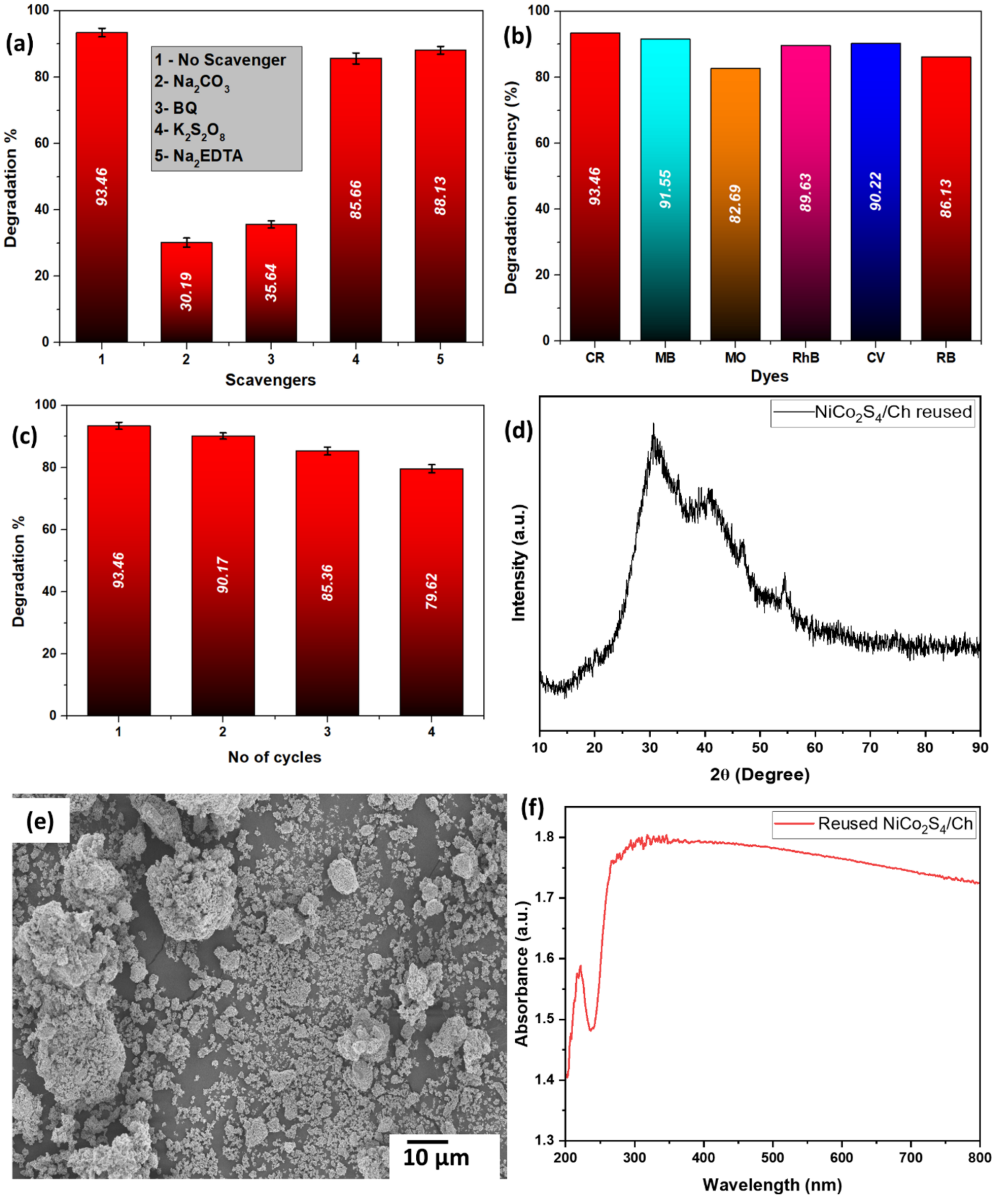
(**a**) Effect of scavenging agents, (**b**) degradation percentage of other dyes, (**c**) reusability performance of NiCo_2_S_4_/Ch, (**d**) XRD spectrum, (**e**) SEM image and (**f**) absorbance spectrum of reused NiCo_2_S_4_/Ch.

The concentration of ROS attacking the target molecules considerably impacts the rate of degradation processes^62^. To examine the role of the photogenerated electrons, holes, OH^∙^ and O_2_^−∙^ radicals on the photodegradation of CR, some sacrificial reagents were added before irradiating the reaction mixture to trap these species and to know which one of them was responsible for the most CR degradation^11^. Sodium carbonate (Na_2_CO_3_) as an OH^∙^ scavenger, benzoquinone (BQ) as an O_2_^−∙^ scavenger, potassium persulfate (K_2_S_2_O_8_) as an e^−^ scavenger and disodium EDTA (Na_2_EDTA) as an h^+^ scavenger were used. As seen in Fig. 11a, BQ and Na_2_CO_3_ significantly retarded the degradation of CR, signifying that O_2_^−∙^ and OH^∙^ played a major role in the degradation of CR dye by NiCo_2_S_4_/Ch nanocomposite. Similar results were also reported in the literature^64^.

### Performance and reusability studies

The photocatalytic performance of NiCo_2_S_4_/Ch nanocomposite for the degradation of other dyes has also been investigated. The photocatalytic degradation of congo red (CR), methylene blue (MB), methyl orange (MO), rhodamine B (RhB), crystal violet (CV) and rose Bengal (RB) were also investigated. As seen in Fig. 11b, the prepared photocatalyst showed excellent photocatalytic activity towards the degradation of these dyes with efficiency greater than 80%, suggesting great potential for the removal of dyes from the aqueous phase.

The recyclability and stability of the synthesized NiCo_2_S_4_/Ch nanocomposite were investigated, and the results are shown in Fig. 11c. The catalyst was centrifuged, washed with an ethanol solution, and dried in the oven at 60 °C for 2 h before being utilized for another cycle. The NiCo_2_S_4_/Ch nanocomposite could be reused and recycled for four successive cycles and showed an efficiency of 79.62 ± 1.23% after the fourth run. A minor decrease in the photocatalytic efficiency was attributed to the blockage of active sites by the degradation products of the CR dye and disruption in the pore structure of the photocatalyst during recycling. The XRD and SEM images of the reused photocatalyst are shown in Fig. 11d,e. The photocatalyst showed no significant change in the phase and morphology. All the peaks corresponding to NiCo_2_S_4_ were visible in the XRD spectrum of the reused photocatalyst. It is worth mentioning that the intensity of the peak of chitosan at 20° was decreased, possibly due to the accumulation of degradation products over the surface of the photocatalyst. However, the SEM image showed the NiCo_2_S_4_ nanoparticles were dispersed over a chitosan sheet, indicating the prepared photocatalyst maintained its structure after four cycles. Additionally, the absorbance spectra of reused NiCo_2_S_4_/Ch photocatalyst (Fig. 11f) showed a slight increase in the intensity in the UV region, which could be due to the adsorption of degradation intermediate products over the surface of the photocatalyst. The high stability and recyclability of the NiCo_2_S_4_/Ch photocatalyst was attributed to the support of the chitosan matrix.

### Mechanism of photodegradation of CR by NiCo_2_S_4_/Ch

**Figure 12:**
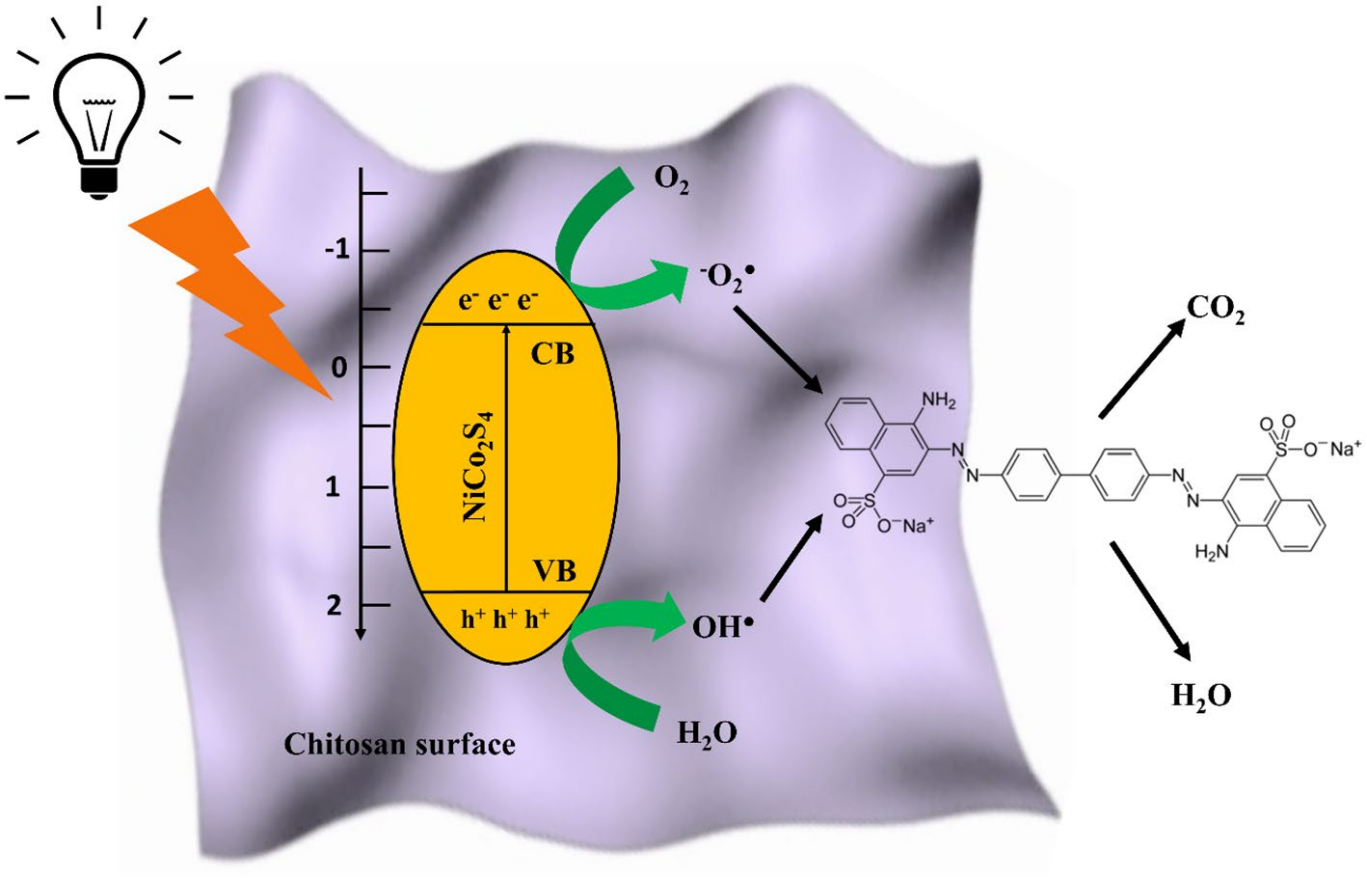
Mechanism of photocatalytic degradation of CR.

The valence and conduction band edge potential of NiCo_2_S_4_ plays a critical role in understanding the photocatalytic degradation mechanism of CR dye. The band positions of NiCo_2_S_4_ were calculated using Mullikan theory^65^. The bandgap and the absolute electronegativity of NiCo_2_S_4_ were calculated to be 2.2 eV^66^. The calculated valance band and conduction band positions of NiCo_2_S_4_ are + 1.93 eV and − 0.27 eV, respectively. A schematic illustration of the photodegradation mechanism of CR dye is shown in Fig. 12. When NiCo_2_S_4_/Ch was irradiated under visible light, the e^−^ are excited from the valance band (VB) to the conduction band (CB) of NiCo_2_S_4_. The excitation of electrons leaves a positive vacancy (h^+^) in the VB. The electron-rich functional groups delocalize the photogenerated holes over the chitosan surface^67^. The photogenerated electrons are absorbed by the oxygen molecules, generating O_2_^−∙^ radicals. Meanwhile, the water molecules absorb the holes, producing OH^∙^ radicals. These radicals initiate a series of photocatalytic reactions attacking the CR molecules and bringing their degradation into simpler molecules^68^. The surface of chitosan and the functional groups helps to retain CR dye molecules over the photocatalyst's surface, while the ROS generated by NiCo_2_S_4_ degrade it. The hydroxyl groups of chitosan strongly attract the positively charged nitrogen of the CR dye^69^. Moreover, chitosan helps in charge of delocalization and enhances photocatalytic efficiency^70^. The photocatalytic reactions responsible for the degradation of CR are as follows^71^:

7 $$Ni{Co}_{2}{S}_{4}/Ch \stackrel{h\nu }{\to } Ni{Co}_{2}{S}_{4}/Ch ({e}^{-}+ {h}^{+})$$

8 $${e}^{-}+{O}_{2}\to {O}_{2}^{-\bullet }$$

9 $${h}^{+}+ {OH}^{-}\to {OH}^{\bullet }$$

10 $${h}^{+}+ {H}_{2}O \to {H}_{2}{O}_{2}+ {H}^{+}$$

11 $${H}_{2}{O}_{2}+{e}^{-}\to {OH}^{-}$$

12 $${H}_{2}{O}_{2}\stackrel{h\nu }{\to } 2{OH}^{\bullet }$$

13 $$CR+ {OH}^{\bullet }+ {O}_{2}^{-\bullet } \to Degradation\, products$$

#### Comparison with other reported photocatalysts

Various materials have been reported to remove CR from the aqueous phase. As prepared, NiCo_2_S_4_/Ch nanocomposite outperformed most of the reported photocatalysts to degrade CR, as shown in Table 3.

**Table 3:** Comparison of NiCo_2_S_4_/Ch with other reported materials for the photodegradation of CR dye.

Photocatalyst	Intial CR concentration (ppm)	Photocatalyst dosage (g/L)	Irradiation source	Time (min)	Degradation (%)	References
Ag_2_WO_4_/Ag_2_S	20	1	200 W Xe lamp	140	99.5	^72^
f–FeO–NC	20	0.2	Sunlight	120	99	^73^
PbTiO_3_	1 × 10^−5^ M	0.5	150 W Xe lamp	150	92	^74^
SrPO NPs	20	1	200 W Xe lamp	210	67	^75^
ZnO	20	0.5	100 W Xe lamp	120	85	^76^
NiCo_2_S_4_/Ch	35	0.44	23 W Philips LED	60	93.46	Present work

## Conclusion

The present study was consistent in assessing the photocatalytic performance of the fabricated NiCo_2_S_4_/Ch nanocomposite and the impacts of different reaction parameters on the photodegradation of CR dye. The effect of pH, photocatalyst dosage, initial dye concentration and irradiation time on the photodegradation of congo red was examined using RSM employing a CCD. According to the ANOVA analysis, a maximum degradation of 93.51% could be attained at the optimum pH of 9, photocatalyst dosage of 22 mg, and CR concentration of 35 ppm in 60 min with a desirability value of 1. Additionally, the statistical parameters like Adj R^2^, Pred R^2^, CV % and Adq precision justified the adequacy of the suggested quadratic model. The synthesized NiCo_2_S_4_/Ch nanocomposite showed a photodegradation efficiency of 93.46% within 60 min under prescribed conditions. The degradation mechanism was studied, and it was found that the composite effectively generated hydroxyl and superoxide radicals that contributed to the photodegradation process. The study emphasizes the possibility of NiCo_2_S_4_/Ch nanocomposite as a recyclable and cost-effective photocatalyst for treating dye-contaminated wastewater.

## Data Availability

All data generated or analyzed during this study are included in this published article.
